# Prevalence and consequences of patient safety incidents in general practice in the Netherlands: a retrospective medical record review study

**DOI:** 10.1186/1748-5908-6-37

**Published:** 2011-04-06

**Authors:** Sander Gaal, Wim Verstappen, René Wolters, Henrike Lankveld, Chris van Weel, Michel Wensing

**Affiliations:** 1 IQ healthcare, Radboud University Nijmegen Medical Centre, the Netherlands, P.O. Box 9101, 6500 HB Nijmegen, the Netherlands; 2 Department of Primary and Community Care, Radboud University Nijmegen Medical Centre, Nijmegen, the Netherlands

## Abstract

**Background:**

Patient safety can be at stake in both hospital and general practice settings. While severe patient safety incidents have been described, quantitative studies in large samples of patients in general practice are rare. This study aimed to assess patient safety in general practice, and to show areas where potential improvements could be implemented.

**Methods:**

We conducted a retrospective review of patient records in Dutch general practice. A random sample of 1,000 patients from 20 general practices was obtained. The number of patient safety incidents that occurred in a one-year period, their perceived underlying causes, and impact on patients' health were recorded.

**Results:**

We identified 211 patient safety incidents across a period of one year (95% CI: 185 until 241). A variety of types of incidents, perceived causes and consequences were found. A total of 58 patient safety incidents affected patients; seven were associated with hospital admission; none resulted in permanent disability or death.

**Conclusions:**

Although this large audit of medical records in general practices identified many patient safety incidents, only a few had a major impact on patients' health. Improving patient safety in this low-risk environment poses specific challenges, given the high numbers of patients and contacts in general practice.

## Background

Since the publication of the landmark report 'To Err is Human' in 1999 [1], patient safety has received considerable attention worldwide, although this attention has been mostly focussed upon hospital care. In countries with a strong primary healthcare system, such as the Netherlands, patients receive most of their medical care in general practice, but to date adequate data on the prevalence of patient safety incidents in general practice are not available [2,3]. In the Netherlands, all citizens are registered with a personal general practitioner (GP), who provides care for a wide range of medical conditions across an extended period of time. About 95% of all presented health problems, which include many chronic and complex diseases, are managed within the general practice setting [4,5]. As shown by Dutch disciplinary law verdicts, very serious and preventable patient safety incidents also occur in primary care [6].

There is no gold standard to identify patient safety incidents [7]. For example, in a pilot study of methods to identify patient safety incidents in primary healthcare, no overlap was found between the different measures of patient safety used in the studies, which included incident reporting, record review, patient questionnaires, and pharmacist-reported events [8]. In the United States, 33 primary care practices (475 clinicians) reported 608 incidents over a two-year period [9]. Another study showed 100 incident reports by healthcare workers in a one year period (with 25,000 visits) in an ambulatory care setting [10]. A prevalence of 5 to 80 adverse events in ambulatory care per 100,000 consultations has been estimated [11]. However, these studies have their limitations. For example, incident reporting by health professionals has not been found to provide valid estimates of the prevalence within a defined setting [8]. Until now, large-scale quantitative studies of patient safety incidents, using random samples of patient records, have only been conducted in hospital settings [12]. The aim of the present study was to determine the prevalence and types of patient safety incidents occurring in general practice in the Netherlands.

## Methods

### Study design and setting

A retrospective medical record review study of 1,000 patients was undertaken to investigate the prevalence of patient safety incidents in general practice in the Netherlands. All procedures and measures were tested in a pilot study and found to be both feasible and reliable [13]. The Dutch Central Committee on Research Involving Human Subjects (CCMO) stated that ethical approval for this study was waived. Each participating practice representative provided formal consent to participate. The reviewers signed a confidentiality agreement to guarantee the privacy of all information. Additional details of the study methods have been published elsewhere [13].

### Sample of patients and practices

A stratified sample of general practices in the Netherlands was adopted in order to obtain a nationally representative sample with regard to practice size and degree of urbanisation. A total of 37 practices were contacted, of which 20 agreed to participate (Table 1). All of the practices included had complete electronic medical records for their patients, which reflects the normal practice situation in the Netherlands. The practices had a total of 72,455 patients and employed a total of 143 healthcare professionals at the time of the study (*e.g.*, GPs or practice nurses). For each practice, 50 patients who visited or contacted the practice between January and March 2009 were randomly selected for inclusion; the records of a total of 1,000 patients were thus reviewed. Patient records were screened from July 2009 onwards, or at least a three-month period after the index contact occurred. This way, potential health outcomes were most likely to become visible, for example through a specialist letter from the hospital. The selection process ensured a proportional spread across the different GPs when more than one GP was working in one of the included practices.

**Table 1:** Practices included

Number of residents in city of practice	
<5000	7
5000 - 30,000	6
30,000 - 100,000	2
>100,000	5
Practice type	
Solo (1 GP)	2
Duo (2 GPs)	4
Group Practice (>2 GPs)	8
Health Centre (also other primary care professions in the same building)	6

Number of GPs in practice	
1	2
2	4
3	3
4	6
7	4
8	1

Average number of patients per practice (SD)	6,433 (2,864)

Practice is a teaching practice for healthcare workers	20

**Patient characteristics**	

Gender	
Male	425
Female	575

Age (%)	
0 to 24	20
25 to 49	32
50 to 74	36
75 to 100	12

Polypharmacy (>5 present medications)	160

Patient at risk	185

Average number of contacts with the practice per year (SD)	8.4 (7.1)

### Definitions

Many definitions of 'patient safety' and 'patient safety incidents' have been published, but these definitions have also been interpreted differently by healthcare professionals [14]. The records of the selected patients from the past 12 months (to review one person year per patient) were reviewed using the following definition of a patient safety incident: 'an unintended event during the care process that resulted, could have resulted, or still might result in harm to the patient' [15]. Only incidents that could have been prevented were looked for in the review, which excluded unintended negative events perceived to be unavoidable.

### Review of patient records

Pilot research showed that the use of a list of triggers to screen the medical records of the 1,000 patients for potential patient safety incidents was not sufficiently sensitive when compared to clinical judgements based upon these records, as was done in comparable studies in hospitals [12,16]. Therefore, all patient records were completely screened by two physicians (SG, HL). When a potential incident was detected, the medical record was printed and reviewed by a third experienced GP (RW). To assess the reliability of this review process, a random sample of 50 patient records was reviewed for potential patient safety incidents by all three of the researchers independently.

### Data analyses

We described the patient safety incidents detected in terms of type of event (organisational, treatment, communication, diagnosis, prevention, or triage), perceived causes of the event (Prevention and Recovery Information System for Monitoring and Analysis: PRISMA method) [17], actual harm caused (international taxonomy of medical errors in primary care) [18], and probability of severe harm. The PRISMA is a root cause analysis tool, which focuses on underlying causes of incidents, and is adopted especially for use in healthcare. Patient safety incidents are described in causal trees and the root causes are classified using the Eindhoven Classification Model (ECM). The ECM divides underlying causes in technical, organisational, human, and other factors. This has been found to produce a reliable classification of the underlying causes of patient safety incidents [17,19]. The Eindhoven Classification Model has also been accepted by the World Alliance for Patient Safety from the World Health Organisation [20,21].

### Statistical analyses

We assumed a normal distribution upon calculating the prevalence of patient safety incidents in Dutch general practice and the associated 95% confidence intervals. An exploratory analysis was conducted on those patient safety incidents with an appreciable effect on patients (*i.e.*, the most serious patient safety incidents). A random coefficient logistic regression model was then applied to determine the effects on such specific patient characteristics as age, gender, polypharmacy, number of practice contacts, patient risk status (*e.g.*, a patient with a history of malignancy, previous myocardial infarction), and the presence of patient safety incidents (*i.e.*, yes/no). Noticeable effects on the patient included a need for extra monitoring, temporary harm, hospital admission, permanent harm, or death.

## Results

The 1,000 patient records included a total of 8,401 patient contacts with the practice. A total of 211 patient safety incidents were identified (95% CI 185 until 241). These incidents concerned 186 patients. In other words, a total of 1 to 4 patient safety incidents per patient were detected per year for a prevalence of 2.2% for all patient contacts (186/8401).

### Agreement between reviewers

The inter-rater reliability showed a κ value of 0.582, and agreement values varied between 82% and 86% for the three reviewers on the presence of a preventable adverse event. This implies that one (not severe) patient safety incident was missed in 50 dossiers. A κ of 0.642 was found for classification of the type of patient safety incidents. With the first given ECM code [13], a κ of 0.736 was found. The severity of harm classification showed a κ of 0.634.

### Types of patient safety incidents

Of the 211 patient safety incidents, 116 were classified as organisation related, 31 as treatment related, 26 as communication related, 21 as diagnostics related, 14 as prevention related, and three as triage related (See table 2 for examples).

**Table 2:** Types of adverse events

Examples of adverse event type	Number (%)
Organisation	
• wrong form was sent with a PAP smear so it could not be evaluated	116 (55.0)
• referral letter was not ready when promised	
• 24 hour blood pressure measurement agreed upon but not performed	

Treatment	
• Patient uses three kinds of antihistaminics	31 (14.7)
• AB prescribed although patient is allergic	
• Too low doses of PPI had been prescribed	

Communication	
• Patient was not told that lab test should be performed on an empty stomach, so had to be repeated	26 (12.3)
• Patient was told to inhale salbutamol (a pulmonary β_2 _adrenergic receptor agonist) prior to the long function test	
• GP agreed to call the patient but forgot	

Diagnosis	
• Recurrent urine infection in a male, without further diagnostics	
• Patient exercise induces shoulder pain, which is considered musculoskeletal; no further research is done; five days later patient is admitted to hospital with a myocardial infarction	21 (10.0)
• Lab result interpreted incorrectly	

Prevention	
• No action on elevated cholesterol in a patient with multiple vascular risk factors	14 (6.6)
• A fasting glucose test was agreed upon, but not performed	
• Administration of NSAID without gastric protection in an elderly patient	

Triage	
• A patient calls with a high fever and pyelonephritis complaints. A home-visit is planned for the next day	3 (1.4)

### Consequences for patients

Of the 211 patient safety incidents, 149 had no tangible effect on the patient (*e.g.*, the GP forgot to call the patient as agreed, an incorrect telephone number was used, or a referral letter was lost). However, a total of 58 events did affect the patient's health or well-being. In four out of the 211 patient safety incidents, the effect on the patient could not be determined. Of the 58 events causing tangible harm, 33 called for extra monitoring of the patient (*e.g.*, extra lab testing, or an extra consult); four caused emotional harm on the part of the patient; 14 caused temporary harm to the patient (*e.g. *fatigue was initially viewed as depression but later found to be associated with a very low haemoglobin); and seven -- out of a total of five patients -- were associated with hospital admission. No patient safety incidents resulting in permanent damage or death were identified (Table 3).

**Table 3:** Consequences of adverse events

Type of error	Number (%)
An error occurred, but the error did not reach the patient.	39 (18.5)
An error occurred that reached the patient, but did not cause the patient harm.	110 (52.1)
An error occurred that reached the patient and required monitoring to confirm that it resulted in no harm to the patient and/or required intervention to preclude harm.	33 (15.6)
An error occurred that may have contributed to or resulted in emotional harm to the patient.	4 (1.9)
An error occurred that may have contributed to or resulted in temporary harm to the patient and required intervention.	14 (6.6)
An error occurred that may have contributed to or resulted in temporary harm to the patient and required initial or prolonged hospitalisation.	7 (3.3)
An error occurred that may have contributed to or resulted in permanent patient harm.	0
An error occurred that required intervention necessary to sustain life.	0
An error occurred that may have contributed to or resulted in the patient's death.	0
An error occurred, but it was not possible to determine harm	4 (1.9)

### Perceived determinants of the patient safety incidents

The causes of the 211 patient safety incidents were analysed through the ECM model, whereupon 348 causes could be identified. Most of the patient safety incidents had a human (50.5%) or an organisational (25.0%) cause. Further analysis of the human causes showed that they mostly concerned wrong coordination of the diagnostic process, a mistaken clinical decision, or errors in the coordination of primary care activities with those of other healthcare professionals. The organisational causes were mostly related to protocols that were not adhered to, or they were culture-based or externally-based. The patient was perceived to have influenced 81 of the patient safety incidents (*e.g.*, not taking a lab test as agreed upon with the physician) (Table 4).

**Table 4:** Underlying causes of adverse events

Main category	Code	Frequency
Technical	External	0
	Design	2
	Construction	1
	Materials	1

Human	External	5
	Clinical decision	29
	Qualifications	1
	Coordination	31
	Verification	18
	Intervention	8
	Guarding the process	84

Organisational	External	16
	Protocols	46
	Knowledge transfer	1
	Management priorities	0
	Culture	24

Patient-related	Patient-related factor	81

Other		0

### Factors associated with incidents

Further analyses showed that the occurrence of patient safety incidents was associated with patient age, polypharmacy, patients at risk (*e.g.*, history of malignancy, history of myocardial infarction), and more than 11 patient contacts per year. In a multivariate model, however, only the number of patient contacts per year remained significant. Those patients who visited the GP more than 11 times a year thus had a higher probability of experiencing a preventable adverse event than other patients (B = 1.313, 95% CI: 0.21 to 2.41).

## Discussion

### Main findings

This study provides an insight into patient safety incidents through medical record review in general practices. A total of 211 patient safety incidents were found to have occurred in 8,401 contacts with the GP practice (in 1,000 patient years). Of these 211 patient safety incidents, 58 affected the patients and seven of these were associated with an unplanned hospital admission.

Other studies of the occurrence of adverse healthcare events reported widely varying prevalence rates. These studies mostly involved incident reporting, although patient reported incidents or malpractice claims have been researched as well. None of these studies undertook a medical record review. Moreover, in our study we only included preventable patient safety incidents, while other studies also included non-preventable incidents. These are important differences, which are likely to yield different numbers and types of incidents. There are also differences between primary care and other sectors, which complicates comparison. In the United States, 33 primary care practices (475 clinicians) reported 608 incidents over a two-year period [9]. Another study showed 100 incident reports by healthcare workers in a one year period (with 25,000 visits) in an ambulatory care setting [10]. A literature review of studies on medical errors in primary care showed a prevalence of 5 to 80 times per 100,000 consultations [11]. The present study showed a much higher rate, namely 2,512 patient safety incidents per 100,000 consultations (95% CI: 2,198 to 2,869). The present findings could reflect the use of a broad definition of the term 'patient safety incident'. In the present study, most (72.5%) of the patient safety incidents indeed had no tangible impact on the health of the patient. If we only consider those patient safety incidents with tangible consequences for the patient, we find a prevalence of 690 patient safety incidents per 100,000 consultations (95% CI: 534 to 891) (0.69% of the patient contacts or in 18.6% per patient per annum), which is still considerably higher than reported in other studies. The large gap between the present data and the numbers published by Sandars in 2003 can be explained in several ways. Sandars' review of the literature mostly included studies that were based upon the reporting of health professionals. While all methods for the measurement of patient safety may involve potential bias [8,22], one could conclude that the direct review of a random sample of medical records could be the most thorough method for the measurement of patient safety incidents. Back in 2003, Sandars also already advised: 'to maximise reliability of error reporting, it is beneficial to obtain data from a second reporter rather than relying on the physician alone.'

The health consequences of the present findings at a national level are potentially quite large. For example, our findings suggest that about 60,000 hospital admissions per year are potentially related or at least partly related to patient safety incidents in primary care (95% CI 25,776 to 140,325). There were 1.8 million hospital admissions in the Netherlands in 2007. This estimate lies within the range of previous studies concerned only with medication errors in the Netherlands and showed 41,000 Dutch hospital admissions per year to be related to medication errors, with 19,000 or almost 50% of these 'severe' medication errors potentially avoidable [12].

From the perspective of the individual patient, however, general practice appears to be safe. Research in hospitals shows one or more patient safety incidents to have occurred in 5.7% of hospital stays, with a preventable adverse event occurring in 2.3% of hospital stays. Other hospital-based studies tend to have even higher incidence rates of approximately 10% [23]. Nevertheless, the occurrence of 1,482 to 2,032 potentially preventable deaths in Dutch hospitals per year is the result of these patient safety incidents in hospitals [12,24]. In contrast, in the present study, no adverse events were found to lead to a preventable death. Although corresponding percentages of patient safety incidents were found in the GP and hospital settings, the potential consequences of the patient safety incidents in general practice were much less serious than those of the patient safety incidents in hospital. This probably reflects the generally lower risk of the majority of interventions conducted in general practice, the fewer number of transfers of patients between health professionals in general practice, and the generally healthier status of patients in the GP setting, as opposed to the hospital care setting.

The results of the present study are of particular relevance to countries with a strong primary care system. About 95% of the health problems of patients in the Netherlands are fully managed by GPs in primary care. The threshold for hospital admission is probably higher compared to countries with less well-developed primary care systems. This could constitute a potential safety risk, as the family practitioner must make clinical decisions with the aid of only a few diagnostic possibilities (*e.g.*, no x-rays, frequently no EKG possibilities). Conversely, this same threshold could actually reduce the risk of iatrogenic damage; fewer false positive test results could occur as a result of less testing in the primary setting and less 'over-testing' of the patient could occur in the primary care setting, compared to the hospital setting. The most serious patient safety incidents in our study were found to be related to clinical decisions in which a 'wait and see' approach was inappropriately adopted. For example, when no further additional testing was conducted for a patient with chest pain. This finding is also in line with the results of other studies that underscore the significance of diagnostic errors [25].

An exploratory analysis of the patient safety incidents showed those patients who visited the primary care practice more than 11 times a year to have a heightened probability of experiencing a preventable adverse event. In a multivariate model, moreover, other variables such as age, gender, polypharmacy, and patient-at-risk lost their significance when included with frequency of practice consult. In other words, the most common health risk factors were not related to the number of patient safety incidents, while frequency of primary care practice visit was. We suggest that the chances of a preventable adverse event are the same for every practice visit, but increased practice visit additively increases the probability of a preventable adverse event due to so-called chance capitalization. One study shows patients with a high frequency of practice visits to be mostly female, have a BMI >30, have alcohol abstinence, and low patient satisfaction, for example [26]. Of course, another -- still unknown -- variable might account for the association.

In our opinion, further research should focus on two points. First, the diagnostic process and the wait and see approach, which is an important tool in general practice, and second, education on patient safety and improvement on this subject.

In sum, serious patient safety incidents appear to have lower prevalence in the general practice than in the hospital setting. Also, the outcomes of patient safety incidents, when they occur, appear to be less serious in the general practice than in the hospital setting. The general practice setting thus appears to be a relatively safe place for the patient, but awareness of harm should nevertheless be enhanced given the potentially detrimental consequences of such harm when it does occur.

### Limitations

Each of the methods available to determine the prevalence of patient safety incidents has its difficulties. The literature shows little overlap in the different methods used to document the prevalence of patient safety incidents [8]. Retrospective studies of patient records currently offer the best means to assess the prevalence of patient safety incidents [22]. Nonetheless, the reporting of patient safety incidents by healthcare professionals may be more appropriate for attaining a more in-depth understanding of patient safety incidents. Even so, many of the reported patient safety incidents stem from organisational and communication problems. There is also a suspicion of underreporting medical errors by healthcare professionals [11]. The generalisability of the present findings could also be limited by the relatively low number of health professionals and primary care practices involved in the study.

The reliability of reviewing patient records could be problematic. In our study however, the inter-rater agreement (κ values) was found to be reasonably good. It thus appears that our level of agreement was comparable, or better than the level of agreement found for similar empirical research conducted in a hospital setting [12,16]. The retrospective interpretation of patient records could nevertheless be biased by hindsight [27].

Finally, in the root cause analyses, we noticed that mostly human and organisational factors played a role in the occurrence of patient safety incidents in primary care. It is known that the underlying causes of patient safety incidents could also be largely technical and system-related [12]. Patient records generally provide insufficient information for a thorough root cause analysis. The present study would therefore have been strengthened if in-depth interviews with family practitioners had been conducted to explore the roles of various contributory factors. This was unfortunately not feasible, due to time and financial constraints.

### Implications for future research

This study provides a much-needed insight into the prevalence of patient safety incidents in Dutch general practice. Few studies have explored the prevalence of adverse effects in this particular healthcare sector, and even fewer studies have done this on the basis of a large-scale analysis of actual patient records. We found only a few patient safety incidents with serious consequences for the patient occurring in general practice. The improvement of patient safety should nevertheless be an ongoing process and thus encouraged.

While we did not find a preventable adverse event in primary care practice to be associated with permanent damage to the patient or death in the analyses of the records of 1,000 patients in the present study, disciplinary law verdicts nevertheless show such patient safety incidents to occur -- also in a primary care setting. The incidence of such severe patient safety incidents in primary care is likely to be very low, which means that a very large number of patient records must be screened to detect these events. This also suggests that not all patient safety incidents find their way into patient records, and that various methods should be adopted in future research to identify all patient safety incidents. Nonetheless, the occurrence of this type of preventable adverse event has an exceptional impact on the individuals involved. Therefore, the occurrence of such a preventable adverse event should never be trivialised.

## Conclusion

A total of 211 patient safety incidents (2.51%) were found to have occurred in 8,401 contacts in general practice, a total of 1,000 patient years. Of these 211 patient safety incidents, 58 were judged to have affected the patients (0.69%). Most of the patient safety incidents found to occur in this setting do not have significant health outcomes for the patient. Nevertheless, serious patient safety incidents can and do occur in general practice as well. Because the majority of patient care has been concentrated in general practice, the net impact of such patient safety incidents could be substantial. Different methods are thus needed to detect and record these patient safety incidents, and it is very important that strategies to improve the safety of general practices also be promoted, as has been done in the hospital setting.

## Competing interests

The authors declare that they have no competing interests.

## Authors' contributions

SG and HL collected the data. SG, HL and RW performed the analyses and presented the results. SG drafted the manuscript. WV, HL, CvW, RW and MW contributed to the conception and design of the study and revised the manuscript. MW supervised the study. All authors read and approved the final manuscript.
